# Prediction of Thermal and Optical Properties of Oxyfluoride Glasses Based on Interpretable Machine Learning

**DOI:** 10.3390/nano15110860

**Published:** 2025-06-03

**Authors:** Yuhao Xie, Xiangfu Wang

**Affiliations:** 1 College of Electronic and Optical Engineering & College of Flexible Electronics (Future Technology), Nanjing University of Posts and Telecommunications, Nanjing 210023, China; 2 College of Integrated Circuit Science and Engineering, Nanjing University of Posts and Telecommunications, Nanjing 210023, China; 3 Yunnan Key Laboratory of Electromagnetic Materials and Devices, Kunming 650091, China

**Keywords:** oxyfluoride glass, machine learning, property prediction, model interpretation

## Abstract

Based on the components of glasses, four algorithms, namely K-Nearest Neighbor, Random Forest, Support Vector Machine, and eXtreme Gradient Boosting, were used to construct an optimal machine learning model to predict the thermal and optical properties of oxyfluoride glass, namely glass transition temperature, density, Abbe number, liquidus temperature, thermal expansion coefficient, and refractive index. We perform SHAP analysis on the constructed machine learning model to explain the effects of different components on the properties. Based on the trained machine learning models, we developed several ternary system prediction maps that can effectively predict the properties of glasses composed of different proportions of components. This study provides a method to design new oxyfluoride glasses only knowing the components of glasses, which is instructive for the development of new types of oxyfluoride glasses as well as for computer-aided reverse design.

## 1. Introduction

As a unique glass material system, oxyfluoride glass is a composite of oxides and fluorides. Compared with traditional oxide glasses, oxyfluoride glasses have unique properties [1,2]. In terms of optics, some oxyfluoride glasses are suitable for working in the ultraviolet spectral region, which can be applied as a laser material; some oxyfluoride glasses enriched with a high concentration of rare-earth ions exhibit high refractive indexes, which is of great significance in the design of optical components. In addition, some oxyfluoride glasses containing rare earth elements have not only unique luminescence properties but also high thermal stability and strong paramagnetism, making them ideal for new optical and magnetic materials.

However, compared with oxide glass, there is an obvious deficiency in the current exploration of the relationship between the composition and properties of oxyfluoride glass. The traditional Edison trial-and-error method requires a large number of experimental attempts, which not only consumes a lot of time but also consumes numerous resources and largely restricts the progress of research on oxyfluoride glass [3]. In recent years, with the development of artificial intelligence technology, researchers have actively introduced machine learning (ML) algorithms, which are dedicated to exploring the intrinsic relationship between composition and properties in the field of glass [4]. In related studies, Ravinder, R., Zaki, M., Cassar, D. R., and others have conducted in-depth studies on various properties of oxide glasses using machine learning algorithms [5,6,7,8,9,10,11,12,13,14,15,16,17,18,19,20,21,22]; Mastelini, S. M., Singla, S., and others have successfully elucidated the correlation between composition and properties of sulfur-based glasses through machine learning [23,24]; Shaik Kareem Ahmmad et al. also attempted to predict the density of fluoride glass with the help of machine learning, but failed to explain the constructed model and did not analyze the important role of fluoride-containing glass systems in optics and other fields [25]. Therefore, the study of composition-property prediction of oxyfluoride glasses through machine learning is of great significance which is expected to accelerate the development of the field of oxyfluoride glass.

In applying machine learning to the field of glass, researchers have explored numerous algorithms, attempted to compare multiple algorithms, and constructed models with optimal performance. Among these algorithms, K-Nearest Neighbors, Random Forest, Neural Networks, Support Vector Machines, and eXtreme Gradient Boosting are commonly used as analytical tools. In the study of oxide glasses, Ravinder, R. used Neural Network modeling to provide insight into the role of 37 oxides in the glass system and developed GSC diagrams [12]. Zaki, M. also used Neural Networks to focus on two key optical properties of glass, refractive index and Abbe number and introduced SHAP analysis to explain the effect of individual oxides on the optical properties [17]. Cassar, D. R. adopted a more comprehensive research strategy, using decision tree induction, K-Nearest Neighbor, and Random Forest algorithms to systematically predict and analyze six properties of oxide glasses [14]. In the study of sulfur glasses, Mastelini S. M., Singla, S., et al. adopted a similar idea to predict, compare, and interpret models for multiple properties of sulfur glasses [23,24]. The composition-property relationships of oxyfluoride glasses are therefore suitable for investigation using these mature machine learning algorithms, which are expected to drive further development in the field.

In this paper, we compare four machine learning algorithms—K-Nearest Neighbors, Random Forest, Support Vector Machines, and eXtreme Gradient Boosting—to construct predictive models for six key properties: glass transition temperature, density, Abbe number, liquidus temperature, thermal expansion coefficient, and refractive index, with respect to the composition-property relationships of oxyfluoride glasses. At the same time, we introduce the Shapley additive interpretation (SHAP) algorithm. Through this algorithm, we explain the effects of each compound composition on the six properties of oxyfluoride glass, thus revealing the intrinsic correlation between the compound compositions and the glass properties. In addition, based on the trained machine learning model, we carry out property prediction for the ternary oxyfluoride glass system. By simulating the prediction of ternary oxyfluoride glass systems with different scales, we develop a series of ternary property prediction diagrams. The plots visualize the properties of the glass in the ternary oxyfluoride glass system with different compositional ratios, which is helpful for the development of new oxyfluoride glasses and computer-aided reverse design.

## 2. Materials and Methods

### 2.1. Data Collection

All glass datasets in this study were constructed based on the processing of the SciGlass database [26] by the Python software package glasspy 0.5.3 [27]. Considering previous data processing methods, we collected statistical information for glasses with a non-zero fluoride percentage under six attributes: glass transition temperature, density, Abbe number, liquidus temperature, thermal expansion coefficient, and refractive index, and implemented the following data processing steps:

1. Ensure that the sum of the glass compositions for each group is exactly 1 in order to circumvent errors that may be introduced by manual preparation.
2. Eliminate redundant data by replacing duplicate entries with median values.
3. Remove extreme values, defined as data points outside the 0.05% and 99.95% percentile ranges. Previous research has shown that some of the extreme values can lead to deterioration in model performance.
4. Removing components with standard deviations less than 10^−3^, i.e., characteristics with very low variance.
5. Apply Variance Inflation Factor (VIF) to remove features with a high degree of multicollinearity.
6. Remove glass with a low fluoride content by setting an appropriate fluoride content threshold for each attribute dataset, a step that allows the model to focus more on the fluoride component and simplifies the number of features, reducing model complexity.
7. Select only those compound components present in at least 10 glass compositions to ensure that the data in the training and test sets are representative.

It is worth noting that when dealing with the dataset of thermal expansion coefficient, we found that the difference in the order of magnitude between the lowest and the highest values of the original dataset amounted to two orders of magnitude, so we took the past literature’s treatment [14] and preprocessed the dataset of this property with a logarithmic function with a base of 10. After the processing, the model performance of this property is greatly improved in three algorithms: KNN, RF, and XGBoost. After the above processing flow, the final statistical results of database information for each property are presented in Table 1.

### 2.2. Machine Learning Algorithms

In this study, we have chosen four algorithms that are commonly used in related research in this field and have excellent performance, namely the K-Nearest Neighbors algorithm (KNN) [28], the Random Forest algorithm (RF) [29], the Support Vector Machines algorithm (SVM) [30] and the eXtreme Gradient Boosting algorithm (XGBoost) [31].

The K-Nearest Neighbors algorithm is based on the principle of local approximation. For a given target sample to be predicted, the algorithm searches for the K neighboring samples that are closest to the target sample in the existing training dataset based on specific distance measures, such as Euclidean distance, Manhattan distance, and so on. Subsequently, the actual output values of these K neighboring samples are integrated by means of weighted averaging, etc., to determine the predicted value of the target sample. In weighted averaging, the closer the neighbor samples are to the target sample, the higher the weight is usually given.

The Random Forest algorithm combines the Bagging technique with Decision Trees. The bagging technique generates multiple distinct sub-datasets by performing a release sampling of the original training dataset. Each sub-dataset is used to train a decision tree independently, and some features are randomly selected during the decision tree construction process to further increase the diversity of the model. Ultimately, the prediction result of the Random Forest is the arithmetic mean of the prediction results of all decision trees. This integration strategy gives full play to the advantages of multiple decision trees and effectively avoids the overfitting problem that is prone to occur in a single decision tree.

The core idea of the Support Vector Machines algorithm is to explore an optimal regression hyperplane in the feature space such that the sum of distances from all sample points to this hyperplane is minimized while allowing for some degree of error. Different from traditional regression methods, SVR introduces the ɛ-insensitive loss function. That is, the loss is not counted for sample points within the “insensitive band” of width ɛ centered on the regression hyperplane; the loss is counted only when the sample points fall outside the “insensitive band”. This feature makes SVR more robust to noise and outliers.

The eXtreme Gradient Boosting algorithm builds powerful regression models by iteratively training a series of decision trees. Each newly generated decision tree is trained based on the prediction errors of all previous trees, with the goal of gradually reducing the overall prediction error. Each decision tree in XGBoost is a regression tree with leaf nodes storing predicted values. During the decision tree construction process, XGBoost uses a greedy algorithm to select features and split points that minimize the loss function each time. By iteratively fitting the gradient and introducing regularization terms, XGBoost is able to efficiently capture complex patterns in the data and has a good tolerance for noise and outliers in the data due to its integrated nature based on multiple decision trees, where the error of a single decision tree has less impact on the overall result.

In our work, we divided the training set and test set in the ratio of 80:20 for training the four algorithm models. Considering that hyperparameters have a significant impact on the performance of the prediction models, we used the grid search method and combined it with a 5-fold cross-validation strategy to optimize the hyperparameters of the four algorithms mentioned above using the R^2^ scores as an evaluation metric [32,33]. In Table 2 and Table 3, we recorded the hyperparameter optimization results of the four algorithms for each attribute. After completing the model optimization, we compared the performance of the four algorithms and constructed the optimal prediction model, on the basis of which we carried out the subsequent SHAP analysis as well as the ternary system prediction.

The algorithms used in this study are provided by Python packages, including NumPy [34], pandas [35], matplotlib [36], Scikit-learn [37], and xgboost. The packages NumPy and pandas are used to process the dataset; matplotlib is used for data visualization; and Scikit-learn and xgboost provide the code for machine learning.

### 2.3. SHAP Analysis

In the field of machine learning, model interpretability is crucial. SHAP, a powerful model interpretation tool whose theory is based on the Shapley value in cooperative game theory, is now widely used in many fields [38,39,40]. In this study, we computed the Shapley value of each glass component with the help of Python’s shap module in order to interpret the six constructed models.

Due to the additivity property of SHAP values, the predicted values of the models can be obtained by adding the SHAP values of all the feature components in a given prediction to a base value, which is usually the mean of the target value. This additivity property provides a clear picture of how much each feature component contributes to the final property prediction and which compounds contribute to the increase or decrease of a given property. In order to visualize the results of the SHAP analysis, we have visualized the top ten components in terms of importance and their positive or negative influence and magnitude on the target property in the form of a beeswarm plot in Section 3.3.

## 3. Results and Discussion

### 3.1. Analysis of the Datasets Used in This Study

**Figure 1:**
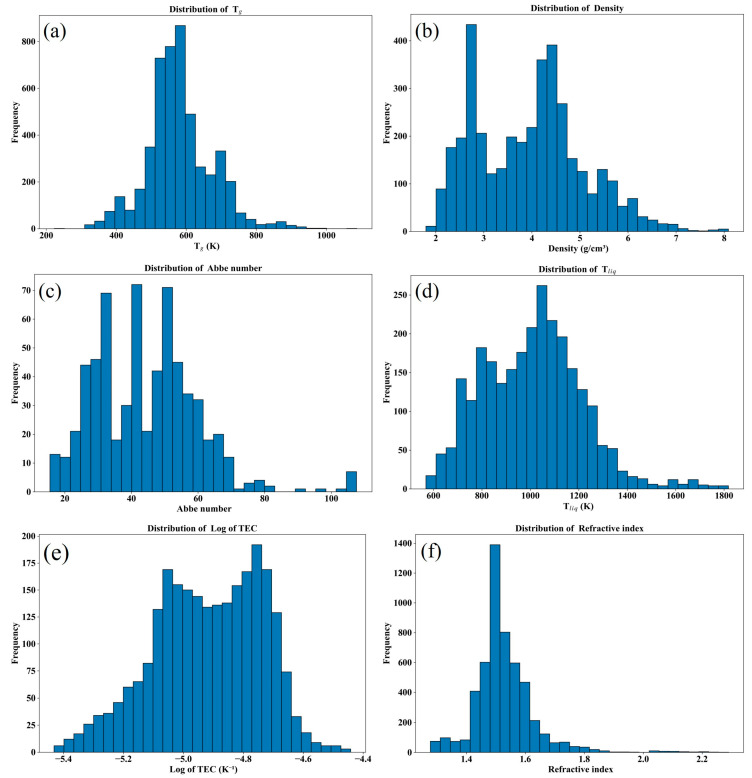
Data distribution of six properties of oxyfluoride glass, (**a**) glass transition temperature, (**b**) density, (**c**) Abbe number, (**d**) liquidus temperature, (**e**) thermal expansion coefficient after taking logarithmic values, (**f**) refractive index.

**Figure 2:**
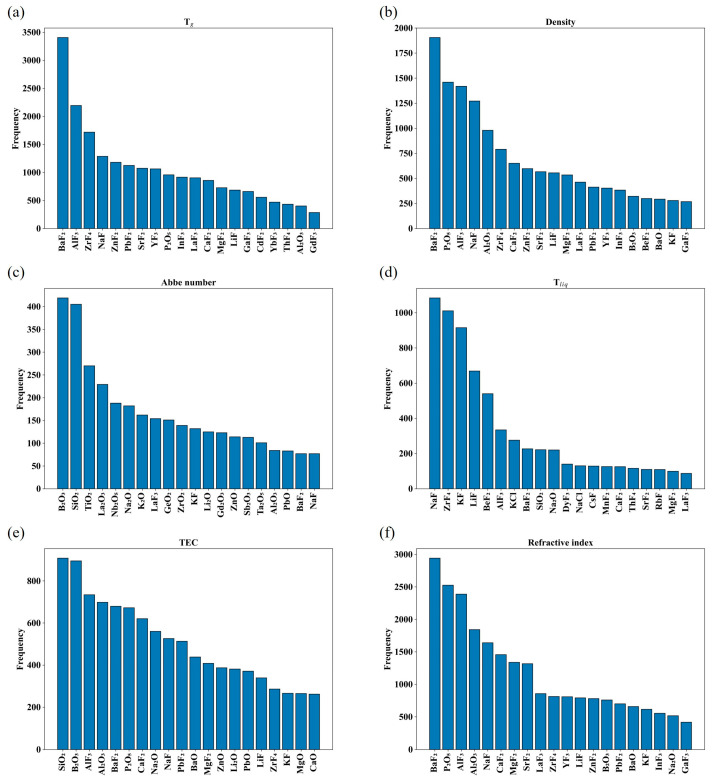
Frequency of occurrence of various compounds for six properties of oxyfluoride glass, (**a**) glass transition temperature, (**b**) density, (**c**) Abbe number, (**d**) liquidus temperature, (**e**) thermal expansion coefficient, (**f**) refractive index.

**Figure 3:**
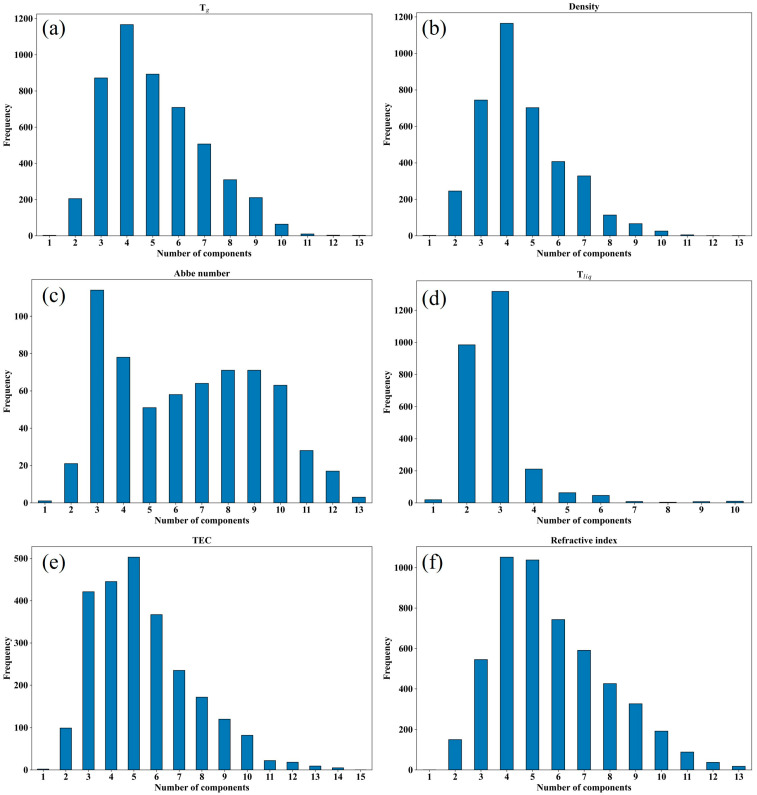
Frequency of the number of glass components for six properties of oxyfluoride glass, (**a**) glass transition temperature, (**b**) density, (**c**) Abbe number, (**d**) liquidus temperature, (**e**) thermal expansion coefficient, (**f**) refractive index.

Figure 1, Figure 2 and Figure 3 and Table 1 summarize a number of statistical metrics for each attribute dataset, including the size of the dataset, the distribution of values, the frequency of occurrence of each compound, and the number of components in each glass. Figure 1 shows the data distribution under each property, and it can be seen that not all properties conform to the normal distribution, which poses a challenge for the optimization and selection of models. Figure 2 shows the number of occurrences of different compounds for each property. In terms of Abbe number, for example, the number of occurrences of B_2_O_3_ and SiO_2_ is high, about 400, much higher than the other compounds. This indicates a certain sparsity in the data set, again challenging the model. Figure 3 shows the number of compounds contained in a group of glasses for each property. As an example, a group of glasses tends to contain no more than 13 compound species in terms of Abbe number. This provides a reference for the development of new oxyfluoride glasses. Table 1 shows the Abbe number is the smallest of the datasets, with 640 instances, while the refractive index dataset is the largest, with 5209 instances. Despite the relatively small size of the dataset for each property compared to the dataset in the oxide glass study, the dataset in this study still shows significant research value and potential for application in the field of machine learning.

Figure 1 shows that the distribution of values for some of the glass properties (e.g., Abbe number and density) is more asymmetric compared to other properties, a phenomenon that may be related to the number of examples available for study. It is worth highlighting that Table 1, Figure 2 and Figure 3 together show the uniqueness of the frequency of occurrence, the number of components, and the number of features considered for each type of compound under each property. This abundance of statistical data provides an extremely rich source of information for glass studies and helps to target specific analyses for different properties.

### 3.2. Predictive Performance Measures

**Figure 4:**
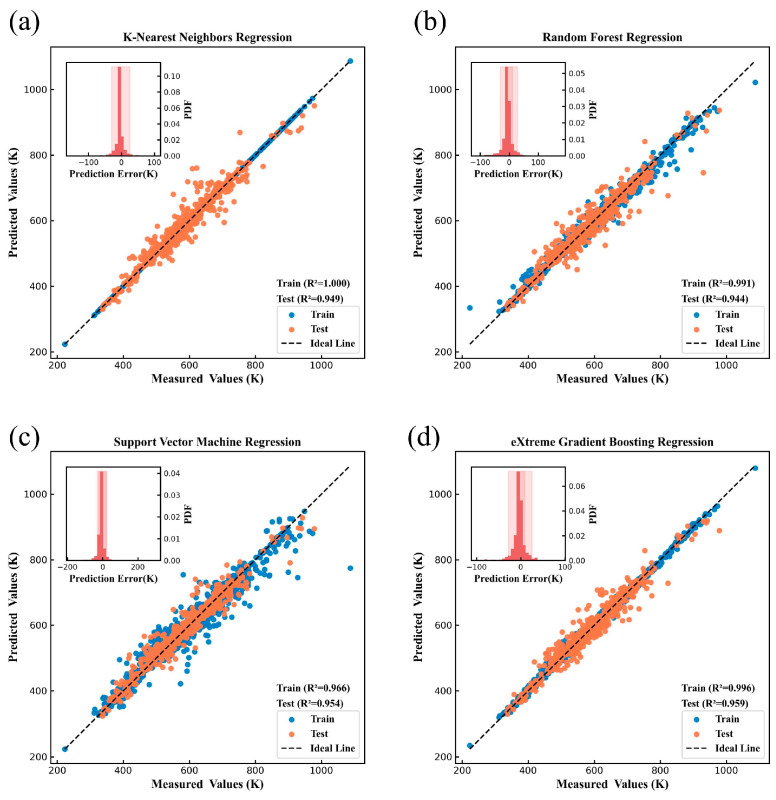
Prediction of glass transition temperature under (**a**) KNN, (**b**) RF, (**c**) SVM, (**d**) XGBoost.

**Figure 5:**
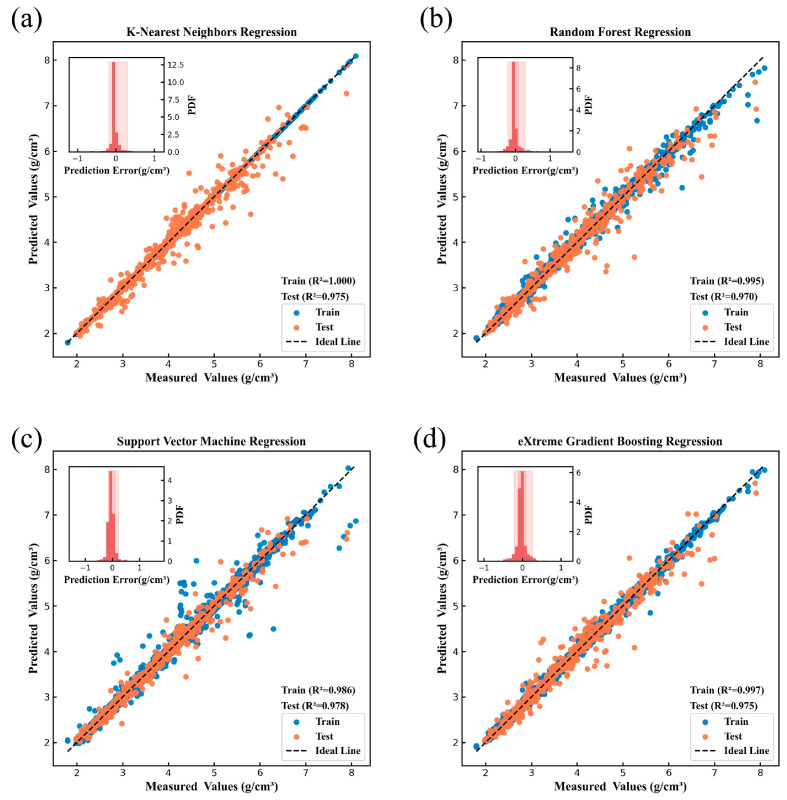
Prediction of density under (**a**) KNN, (**b**) RF, (**c**) SVM, (**d**) XGBoost.

**Figure 6:**
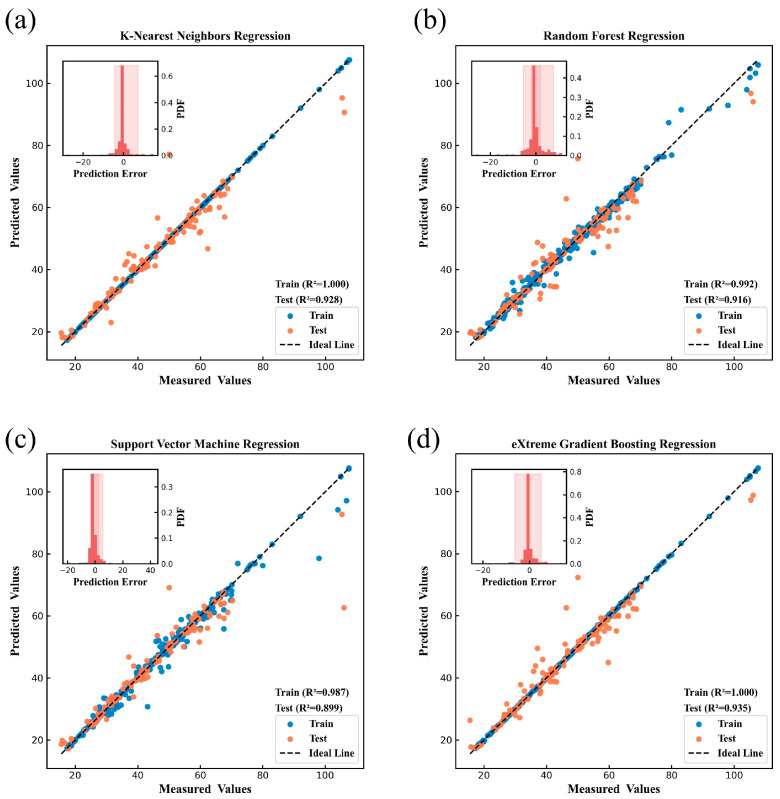
Prediction of Abbe number under (**a**) KNN, (**b**) RF, (**c**) SVM, (**d**) XGBoost.

**Figure 7:**
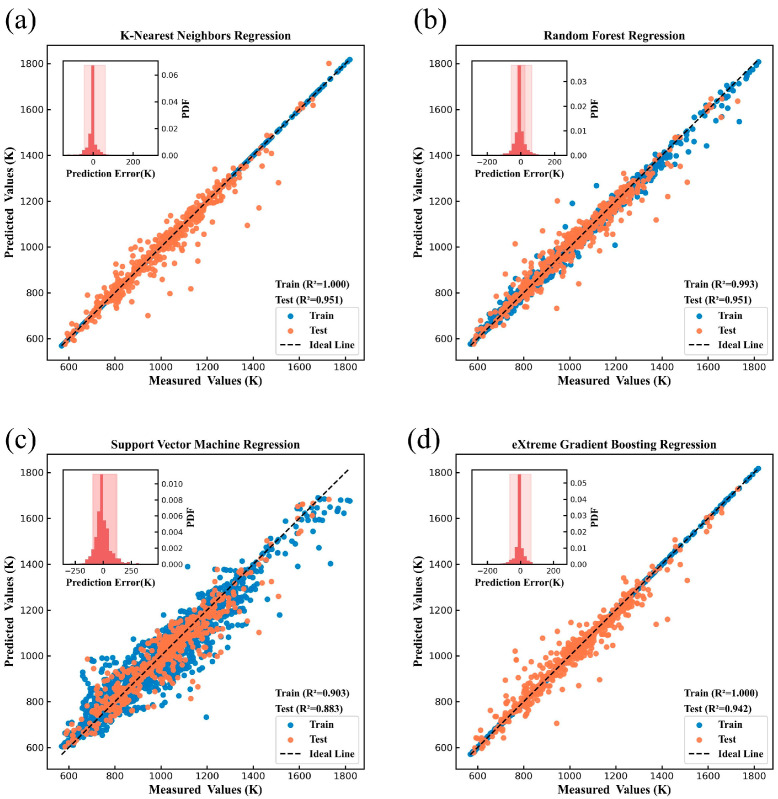
Prediction of liquidus temperature under (**a**) KNN, (**b**) RF, (**c**) SVM, (**d**) XGBoost.

**Figure 8:**
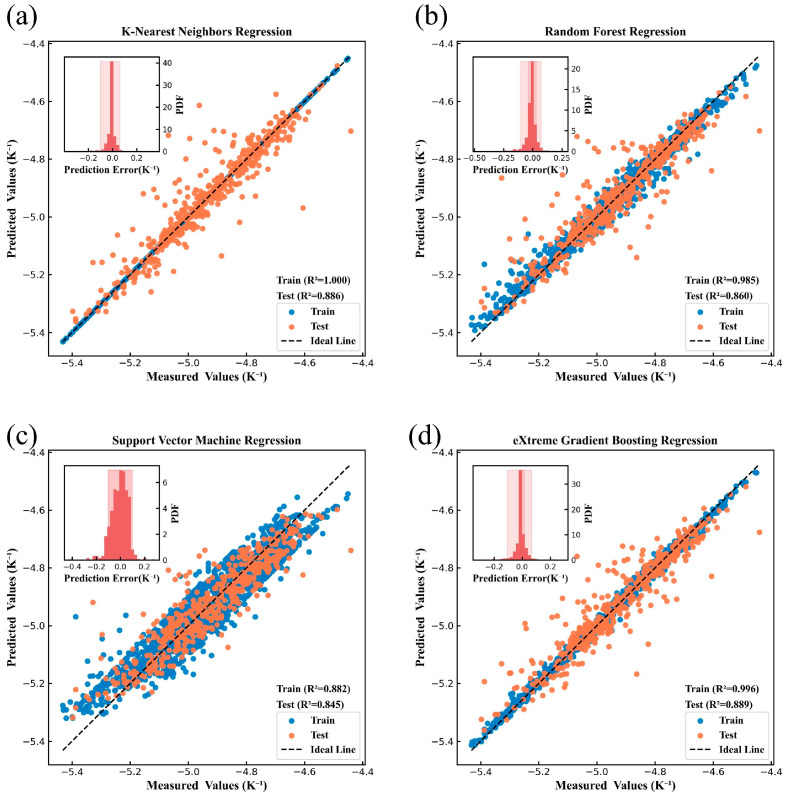
Prediction of thermal expansion coefficient under (**a**) KNN, (**b**) RF, (**c**) SVM, (**d**) XGBoost.

**Figure 9:**
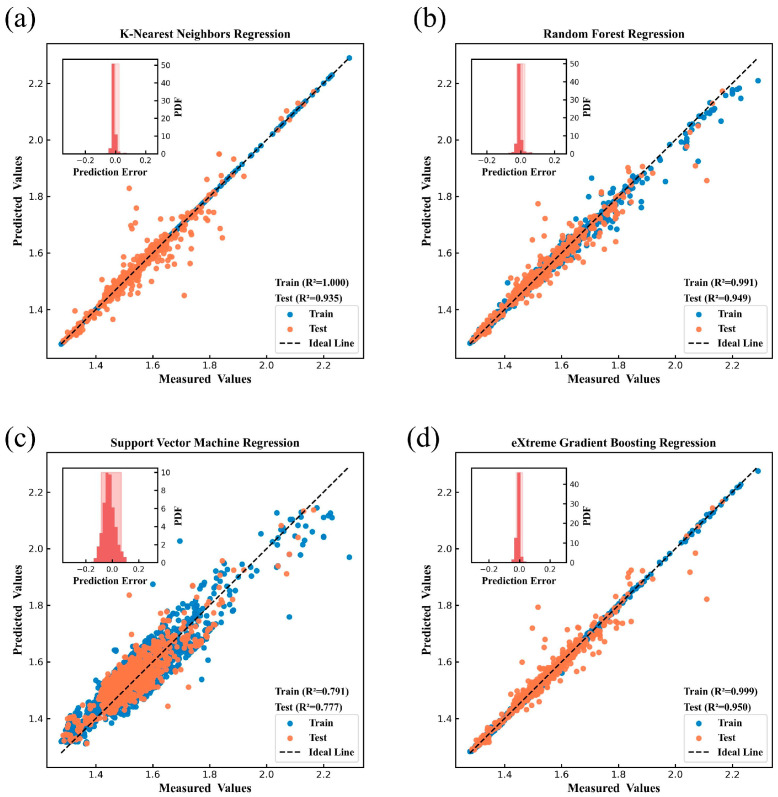
Refractive index prediction under (**a**) KNN, (**b**) RF, (**c**) SVM, (**d**) XGBoost.

Figure 4, Figure 5, Figure 6, Figure 7, Figure 8 and Figure 9 show the subplots of the predicted and experimental deviations, R^2^ scores, and probability density functions of the errors (insets located in the upper left corner of the figures) for the six properties: glass transition temperature, density, Abbe number, liquidus temperature, thermal expansion coefficient, and refractive index for the four algorithms on both the training and test sets. It is important to note that the four algorithms are carefully hyper-parameterized for each attribute (the hyper-parameters are recorded in Table 2 and Table 3), and thus, the optimal model for each algorithm is presented in the figure. Meanwhile, a 45-degree straight line representing the ideal state is marked in each figure, and the closer the data points are to this line, the closer the predicted values are to the experimental values and the better the prediction effect of the model is. In addition, the probability density function (PDF) of the errors and their corresponding 90% confidence intervals (presented on a light background) are attached in the upper left corner of each figure to visualize the distribution of the errors of the algorithms. We also count the training time and other performance metrics, including MAE and RMSE, of each optimized model in Table 4 and Table 5, which allow us to visually and comprehensively observe the differences between different algorithms.

In the field of machine learning, the R^2^ score is used as a widely used evaluation metric to measure the proportion of the variance in the predicted variable that can be explained by the characteristics of the independent variable and the model. The closer its value tends to 1, it means the better the model fits the data and the better the model’s explanatory and predictive performance. MAE (Mean Absolute Error), as an intuitive measure of error, directly reflects the average level of prediction deviation by calculating the mean of the absolute difference between the predicted value and the true value, is insensitive to outliers, is robust, and facilitates an intuitive understanding of the actual size of the prediction error. RMSE (Root Mean Square Error) focuses on the measurement of the degree of dispersion of the predicted value by taking the root of the square of the error, highlighting the effect of extreme errors, and can sensitively reflect the model’s ability to deal with outliers.

Figure 4, Figure 5, Figure 6, Figure 7, Figure 8 and Figure 9, Table 4 and Table 5 show that different algorithms can achieve different optimal performance on datasets with different attributes and can construct the best model for the attributes. In terms of glass transition temperature, XGBoost has the best performance, followed by SVM. In terms of density, all four algorithms show high performance, with KNN and SVM standing out. In terms of Abbe number, XGBoost shows the best performance, followed by KNN. In terms of liquid phase temperature, KNN and RF show the best performance. In this study, the coefficient of thermal expansion is the more difficult property to predict, and the four algorithms perform slightly lower than the other properties in this property, with KNN showing the best performance, followed by XGBoost. In refractive index, XGBoost and RF perform well. It is also worth noting that in terms of training time, KNN has a significant advantage, XGBoost and SVM are moderate, and RF consumes the longest time, which requires extra attention when dealing with larger and more complex datasets.

Overall, the KNN algorithm is simple and efficient, with outstanding performance in the prediction of multiple properties, but the algorithm itself has low complexity and weak ability to capture nonlinear relationships; RF improves the model complexity through integrated learning, but the training time is relatively long, and it is weak in dealing with high-dimensional sparse datasets; the SVM algorithm is robust, and it performs well in the prediction of some properties, but the algorithm requires higher requirements for the selection and optimization of the kernel function; XGBoost algorithm is relatively more complex, but the training time is moderate, and the performance achieved is outstanding in almost all properties. Therefore, we choose the model constructed based on XGBoost for the subsequent research work to ensure the consistency of the evaluation system in the subsequent SHAP analysis and ternary system prediction process.

### 3.3. Interpreting the Induced Models

**Figure 10:**
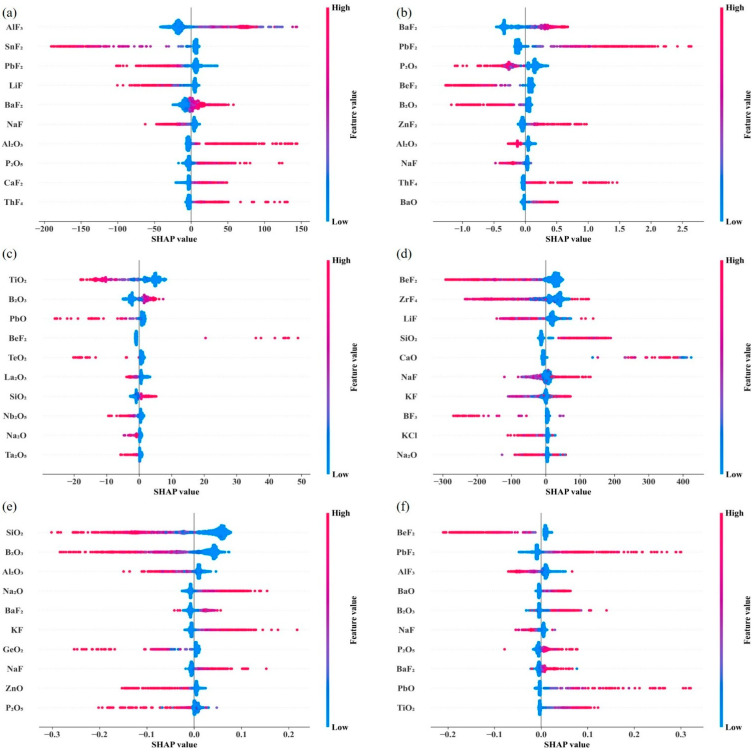
Beeswarm plot investigating SHAP values for six properties. (**a**) glass transition temperature, (**b**) density, (**c**) Abbe number, (**d**) liquidus temperature, (**e**) thermal expansion coefficient, and (**f**) refractive index. The labels on the left show the ten most important compounds identified by SHAP analysis in decreasing order of importance.

As mentioned above, we applied the SHAP algorithm to describe the optimal models we constructed for the six properties and analyzed the contribution of each compound to the predicted properties [41]. In Figure 10, we visualize the top ten compounds in terms of importance in each property in the form of beeswarm plots, ranked from top to bottom according to the level of importance.

With the help of these beeswarm plots, researchers were able to qualitatively analyze the effect of compounds on properties. Taking the glass transition temperature as an example, the components can be categorized into two groups based on the direction of their influence on the predicted values: those that contribute to an increase in the predicted values, including AlF_3_, BaF_2_, Al_2_O_3_, P_2_O_5_, CaF_2_, ThF_4_, and those that lead to a decrease in the predicted values, which are, in order of importance, SnF_2_, PbF_2_, LiF, and NaF. It is worth noting that the importance of the components changes across the different properties analyzed, and some of the components may show a shift from increasing to decreasing model output. In addition, the synergistic effect of components on multiple properties should not be overlooked. For example, the presence of BeF_2_ causes a decrease in the values of density, liquidus temperature, and refractive index, while NaF drives an increase in the thermal expansion coefficient while attenuating the glass transition temperature and density. This complex relationship needs to be emphasized in the development of new oxyfluoride glass designs as well as in computer-aided reverse design.

### 3.4. Model Application

**Figure 11:**
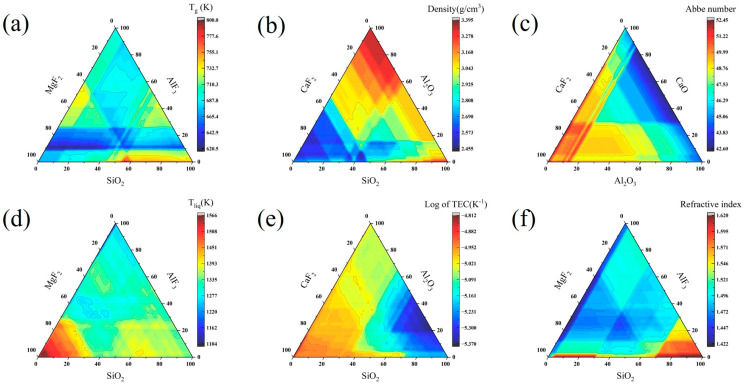
Ternary diagrams for different systems: (**a**) glass transition temperature of MgF_2_, AlF_3_, and SiO_2_ systems, (**b**) density of CaF_2_, Al_2_O_3_, and SiO_2_ systems, (**c**) Abbe number of CaF_2_, CaO, and Al_2_O_3_ systems, (**d**) liquidus temperature of MgF_2_, AlF_3_, SiO_2_ systems, (**e**) thermal expansion coefficient (logarithmic form) of CaF_2_, Al_2_O_3_, SiO_2_ systems, (**f**) refractive index of MgF_2_, AlF_3_, SiO_2_ systems.

To further explore the model performance, Figure 11 shows the ternary diagrams of different oxyfluoride glass systems [42,43,44], which cover the prediction results of glass transition temperature, density, Abbe number, liquidus temperature, thermal expansion coefficient, and refractive index. As seen from the plots, the properties of oxyfluoride glass vary with the ratio of different components, which can help to rationalize the design of the composition of oxyfluoride glass. Taking liquidus temperature as an example, when a high liquidus temperature is required, the proportion of MgF_2_ in the glass should be controlled at 85–95%, AlF_3_ at 0–10%, and SiO_2_ at 0–10%; if a low liquidus temperature is expected, the proportion of MgF_2_ in the glass should be controlled at 0–10%, AlF_3_ at 80–90%, and SiO_2_ at 0–10%. It can be seen that the model is capable of making predictions for the complete compositional domain, finding better solutions for the requirements, and exploring new types of glasses that have not been exploited so far.

However, it is important to note that some of the components shown in the ternary diagrams may not be able to form glass under actual experimental conditions. Therefore, in regions with missing or sparse data points, the predictions of the model may not be reliable. In this case, expert knowledge plays a crucial role in analyzing the validity of the predictions. Even so, the model shows a strong potential for application in the field of computer-aided design and can provide technical support for the design and development of oxyfluoride glass.

## 4. Conclusions

In this research work, we collected an extensive dataset of oxyfluoride glasses and carried out the training and study of four machine-learning algorithms for six glass properties. Specifically, we employed K-Nearest Neighbors, Random Forests, Support Vector Machines, and eXtreme Gradient Boosting algorithms to construct prediction models for glass transition temperature, density, Abbe number, liquidus temperature, thermal expansion coefficient, and refractive index, respectively. In order to achieve the optimal performance of each model, we applied the grid search method to adjust the model hyperparameters. The results show that the XGBoost algorithm exhibits overall excellent performance in the task of predicting the above six glass properties, and the rest of the algorithms perform well for some of the properties. In addition, with the help of the SHAP algorithm, we have explained the effect of individual components on the properties. This can help in the development of new oxyfluoride glasses as well as computer-aided reverse design. Finally, we have predicted the properties of oxyfluoride glass for different ternary systems. Although the relatively limited number of dataset instances used to train the model results in a dip in the model’s performance in the data-sparse region, our model is still important for screening or designing novel glass materials with the right combination of properties. In summary, our study bridges the gap of composition-property relationship in the field of oxyfluoride glass through machine learning and can also serve as a reference for the study of other glass systems.

## Figures and Tables

**Table 1:** Descriptive statistics of the dataset used.

	T_g_	Density	AbbeNumber	T_liq_	Log_10_(TEC)	RefractiveIndex
Number of oxides	29	38	27	11	39	41
Number of fluorides	43	41	19	28	29	48
count	4955	3806	640	2669	2502	5209
max	1087.15	8.09	107.6	1818.15	−4.44	2.29
min	223.15	1.79	15.53	569.15	−5.43	1.27
mean	580.46	3.95	44.35	1009.56	−4.91	1.52

**Table 2:** Hyperparameters of the algorithms used for glass transition temperature, density, and Abbe number.

Model	Hyperparameter	T_g_	Density	AbbeNumber
KNN	n_neighbors	3	3	3
	p	1	1	1
	weights	distance	distance	distance
RF	max_depth	none	none	none
	min_samples_leaf	1	1	1
	min_samples_split	2	2	4
	n_estimators	200	400	400
SVM	C	100	100	100
	gamma	scale	1	scale
	kernel	poly	rbf	rbf
XGBoost	colsample_bytree	0.9	0.8	0.9
	learning_rate	0.1	0.1	0.05
	max_depth	9	7	11
	n_estimators	300	300	300
	subsample	0.9	0.8	0.8

**Table 3:** Hyperparameters of the algorithm used for liquidus temperature, thermal expansion coefficient, and refractive index.

Model	Hyperparameter	T_liq_	Log_10_(TEC)	RefractiveIndex
KNN	n_neighbors	3	3	3
	p	1	1	1
	weights	distance	distance	distance
RF	max_depth	none	none	none
	min_samples_leaf	1	1	1
	min_samples_split	2	2	2
	n_estimators	300	400	300
SVM	C	100	1	100
	gamma	scale	scale	0.1
	kernel	rbf	rbf	rbf
XGBoost	colsample_bytree	0.9	0.8	1
	learning_rate	0.2	0.05	0.2
	max_depth	11	11	7
	n_estimators	300	300	300
	subsample	0.8	0.9	0.9

**Table 4:** The training time of each optimized model (unit: seconds).

	T_g_	Density	AbbeNumber	T_liq_	Log_10_(TEC)	RefractiveIndex
KNN	0.0010	0.0010	0.0010	0.0010	0.0010	0.0010
RF	7.7798	6.9369	2.0694	4.3618	8.2211	16.2200
SVM	1.5781	0.5985	0.0380	0.1833	0.0370	0.0812
XGBoost	0.6149	0.3840	0.4191	0.5659	0.9105	0.5850

**Table 5:** The performance of different algorithms under various evaluation indicators.

		T_g_	Density	AbbeNumber	T_liq_	Log_10_(TEC)	RefractiveIndex
R^2^	KNN	0.949	0.975	0.928	0.951	0.886	0.935
	RF	0.944	0.970	0.916	0.951	0.860	0.949
	SVM	0.954	0.978	0.899	0.883	0.845	0.777
	XGBoost	0.959	0.975	0.935	0.942	0.889	0.950
MAE	KNN	11.588	0.094	2.223	25.111	0.034	0.011
	RF	12.290	0.102	2.652	27.253	0.038	0.011
	SVM	11.150	0.094	2.070	46.643	0.052	0.037
	XGBoost	10.939	0.097	2.176	29.622	0.036	0.010
RMSE	KNN	20.104	0.176	4.189	44.177	0.059	0.026
	RF	21.149	0.192	4.530	44.365	0.065	0.023
	SVM	19.094	0.167	4.980	68.118	0.068	0.048
	XGBoost	18.024	0.175	3.982	47.863	0.058	0.023

## Data Availability

Data are contained within the article.
